# Identification of a pleiotropic effect of *ADIPOQ* on cardiac dysfunction and Alzheimer’s disease based on genetic evidence and health care records

**DOI:** 10.1038/s41398-022-02144-0

**Published:** 2022-09-16

**Authors:** Hyojung Paik, Junehawk Lee, Chan-Seok Jeong, Jun Sung Park, Jeong Ho Lee, Nadav Rappoport, Younghoon Kim, Hee-Young Sohn, Chulman Jo, Jimin Kim, Seong Beom Cho

**Affiliations:** 1 Division of Supercomputing, Korea Institute of Science and Technology Information, Daejeon, 34141 Republic of Korea; 2 Bakar Computational Health Sciences Institute, University of California San Francisco, 550 16th Street, San Francisco, CA 94143 USA; 3 Department of Pediatrics, School of Medicine, University of California San Francisco, 550 16th Street, San Francisco, CA 94143 USA; 4 Department of Data and HPC Science, University of Science and Technology, Daejeon, 34113 Republic of Korea; 5 Biomedical Science and Engineering Interdisciplinary Program, Korea Advanced Institute of Science and Technology (KAIST), Daejeon, 34141 Republic of Korea; 6 Graduate School of Medical Science and Engineering, Korea Advanced Institute of Science and Technology (KAIST), Daejeon, 34141 Republic of Korea; 7 Departement of Software and Information Systems Engineering, Ben-Gurion University of the Negev, Beersheba, 8410501 Israel; 8 Division of Brain Disease Research, Department for Chronic Disease Convergence Research, Korea National Institute of Health, Chungcheongbuk-do, 28159 Republic of Korea; 9 Department of Bio-Medical Informatics, Gachon University, College of Medicine, Incheon, 21565 Republic of Korea

**Keywords:** Personalized medicine, Prognostic markers

## Abstract

Observations of comorbidity in heart diseases, including cardiac dysfunction (CD) are increasing, including and cognitive impairment, such as Alzheimer’s disease and dementia (AD/D). This comorbidity might be due to a pleiotropic effect of genetic variants shared between CD and AD/D. Here, we validated comorbidity of CD and AD/D based on diagnostic records from millions of patients in Korea and the University of California, San Francisco Medical Center (odds ratio 11.5 [8.5–15.5, 95% Confidence Interval (CI)]). By integrating a comprehensive human disease–SNP association database (VARIMED, VARiants Informing MEDicine) and whole-exome sequencing of 50 brains from individuals with and without Alzheimer's disease (AD), we identified missense variants in coding regions including *APOB*, a known risk factor for CD and AD/D, which potentially have a pleiotropic role in both diseases. Of the identified variants, site-directed mutation of *ADIPOQ* (268 G > A; Gly90Ser) in neurons produced abnormal aggregation of tau proteins (*p* = 0.02), suggesting a functional impact for AD/D. The association of CD and *ADIPOQ* variants was confirmed based on domain deletion in cardiac cells. Using the UK Biobank including data from over 500000 individuals, we examined a pleiotropic effect of the *ADIPOQ* variant by comparing CD- and AD/D-associated phenotypic evidence, including cardiac hypertrophy and cognitive degeneration. These results indicate that convergence of health care records and genetic evidences may help to dissect the molecular underpinnings of heart disease and associated cognitive impairment, and could potentially serve a prognostic function. Validation of disease–disease associations through health care records and genomic evidence can determine whether health conditions share risk factors based on pleiotropy.

## Introduction

Adequate identification and risk stratification of patients based on substantial evidence including genetic contributions is a central theme in precision medicine [1]. For example, the identification of a pleiotropic effect as a risk factor shared between diseases is a central premise for beneficial care outcomes including co-occurring (or sequential) diagnosis of diseases in an individual [2, 3], and for predicting mortality [4]. While mapping disease relationships has a long history, the advent of digitalized health records has led to a rapid increase in ability to analyze health care data. This allows for the recapitulation of known temporal disease correlations, such as illness trajectories in Denmark [5]. For example, in our previous study, we traced sequential diagnosis patterns of millions of patients, then identified an unexpected risk of patients with schizophrenia that would change clinical care meaningfully [6]. However, the validation of newly identified genomic risk factors underpinning the comorbidity between diseases using functional evidence has shown limited success.

Pleiotropy is a phenomenon whereby one gene affects multiple traits and appears to be pervasive in biology. Over decades, genome-wide association studies (GWAS) have illustrated that trait-associated genetic variants are substantially shared across different traits, which may lead to comorbid diseases [7]. Detection of shared genetic risks between traits and diseases have been mainly presented based on a large-scale analysis of GWAS data [8]. Li et al. determined that corpuscular volume is elevated before diagnosis of acute lymphoblastic leukemia by associated variants in the gene for IKZF1 through the integration of electronic medical records (EMRs) and a comprehensive GWAS database, VARIMED [9]. In other previous attempts, the beneficial results of clinical application of analysis of pleiotropy effect have been demonstrated via subtyping of patients with type 2 diabetes using their personal genotypes and aligning medical records, including those for disease comorbidities [10].

Dementia is a spectrum of neurological conditions and is an increasingly prevalent issue, covering a wide range of medical conditions, including Alzheimer's disease (AD), dementia with Lewy bodies, and vascular dementia. The risk factors for Alzheimer's disease, and dementia (AD/D) have been suggested in previous attempts to identify them, which are mainly based on the analysis of clinical epidemiology, such as smoking pattern [11] and hemodynamic monitoring [12]. Various etiologies of cardiac dysfunction (CD), such as heart failure, obesity, and myocardial infarction (heart attack), have been ranked [13]. Multimorbid conditions (i.e. multiple diseases in a single patient) are a major clinical issue in patients with CD [14, 15]. Interestingly, patients with CD present a higher risk for AD/D [16]. Although it is suspected that heart disease and AD/D share similar genetic backgrounds and risk factors, such as ApoE polymorphisms [17], many aspects of the genetic architecture shared between AD/D and CD remain unknown.

In this study, we recapitulate the comorbidity of CD and AD/D by repurposing the digital health records of a large number of patients from different countries. The purpose of this is to unravel the shared genetic risk factors of these diseases based on functional evidence, which comprises a genetic associations database, in vitro experiments, medical images, and associated cognitive behavior.

## Methods

### Use of administrative health care records

The dataset used in this analysis was the National Inpatients Set of Korea (NISK) from the Health Insurance Review and Assessment Service (HIRA; www.hira.or.kr). The HIRA database contains deidentified inpatient and outpatient billing information from all medical facilities in Korea covered by insurance, including primary care and academic medical centers. We used three of the annual builds of HIRA (2009–2011) and merged these iterations. To avoid redundancy in the merged HIRA dataset, we only used records of deceased individuals and their diagnostic records in the 2009–2010 versions, and merged these with the most recent 2011 version. All diagnostic codes were reported using the International Classification of Disease, 10th Revision (ICD-10) and grouped to the 3-letter code level to minimize overlap and subclassification of diagnoses. To validate the identified diagnostic patterns, we utilized electronic medical records (EMRs) from the University of California, San Francisco (UCSF) Medical Center, collected using Epic (Verona, WI) between 2011 and 2017, which includes inpatient and outpatient records for 816504 unique individuals. The records were deidentified and contained no direct patient identifiers as defined by the Health Insurance Portability and Accountability Act (HIPAA).

### Disease trajectory (temporal diagnoses of diseases) by directed acyclic graph modeling

The primary diagnosis code was used to determine the primary disease state of each patient. We used a method identical to that used in our previous work to trace the temporal order of disease diagnoses for each patient [6, 18]. In summary, a trajectory consists of the first node for patients diagnosed as being with disease *i* and nodes for subsequent diagnoses presenting in patients who were diagnosed in a prior diagnosis node, which are connected via directed acyclic edges showing that subsequent diagnoses occurred more frequently than at random. To determine the association between a pair of diseases that were co-diagnosed in one patient, we first calculated the relative association (RA) measurement of all disease pairs (*Disease i* – *Disease j*) that occurred within 1 year for each patient. Then, we quantified the overall temporal directionality of disease co-occurrence (*δ*_i→j_ for *Disease i* → *Disease j*) using mean difference of dates of diagnoses or associated admissions for each individual. The statistical significance was determined by a binomial test (Benjamin–Hochberg false discovery rate [FDR] < 0.1). Finally, we used selected pairs of disease co-occurrences (RA > 1, FDR < 0.1) with the directional order of diagnoses (*δ*_i→j_ ≠ 0, FDR < 0.1) in further analysis.

Based on the shared patients between two pairs of diagnoses steps (Disease 1→2 and Disease 2→3, FDR < 0.1), we joined multiple steps of disease-to-disease trajectories by concatenating the progressions of the two diseases into three or multiple steps of diseases (Disease 1→2→3). All details of methods utilized are presented in Additional file 1.

### Analysis of whole-exome sequencing of AD/D samples

#### Tissue collection and genomic DNA extraction

In the present study, we used the same samples and case-control criteria as used in our previous work [19]. In summary, two brain banks (the Netherlands Brain Bank [NBB] and the Human Brain and Spinal Fluid Resource Center [HBSFRC]) provided fresh frozen human postmortem hippocampus and matched whole blood samples. Samples were classified into AD-affected, and unaffected age-matched controls based on clinical and neuropathological findings. Genomic DNA (gDNA) was isolated from 6 to 10 Nissl-stained tissue slides by laser capture microdissection (LCM). Cryosectioning of a frozen hippocampal tissue block (20 μm depth) was performed by cryostat (CM1850, Leica Germany) and attached to ultraviolet (UV)-treated 1.0 mm PEN-membrane slides (Zeiss, 415190-9041-000). The slides were stained with 1% cresyl violet-75% EtOH solution right before LCM. After confirming the proper staining of sub-regions of the hippocampal formation (HIF), slides were mounted on the stage of an LCM instrument (PALM MicroBeam, Zeiss Germany). HIFs from the slides were captured and stored in a lysis buffer (56304, Qiagen, Germany). Mechanical crushing of the acquired tissue was performed using a bead-beating homogenizer (FastPrep-24, MP Biomedicals USA). The homogenized tissue was then lysed at 56 °C for 12 h, using a column-based QIAamp DNA Micro Kit (56304, Qiagen). Whole blood cells were processed using QIAamp DNA Blood Midi Kit (51183, Qiagen), according to the manufacturer’s instructions. Quantification of extracted gDNA was performed using a Bioanalyzer 2100 (Agilent, USA), and its integrity was checked by running it on 1% agarose gel. The volume of the LCM-captured region from the PALM Robo software and the concentration of gDNA from the Bioanalyzer was used to determine the average gDNA yield from the HIF. The process of estimating the number of neurons in the LCM-captured region is as follows: first, the average number of observed neurons in each sub-region of the HIF is determined by finding the number of maximum signals from 8-bit converted Nissl-stained images using ImageJ software. Thereafter, the area of the LCM-captured region is multiplied by the average neuron count.

#### Whole-exome sequencing

Each exome library was prepared based on the manufacturer’s instructions (Human All Exon V4/V5 + UTR 50 Mb Kit, Agilent USA) using about 1 μg of gDNA. Paired-end sequencing with an Illumina HiSeq 2000/2500 sequencing system was performed according to the manufacturer’s protocol using quality control-passed exome libraries (http://nextgen.mgh.harvard.edu/attachments/Paired-End_SamplePrep_Guide_1005063_D.pdf).

To identify germline variants from the samples, we applied the GATK’s best practice workflows. First, germline variants of each sample were identified with a GATK HaplotypeCaller (release 3.5.0) in GVCF mode. All GVCFs generated from each sample were jointly genotyped by using the GATK GenotypeGVCFs tool (release 3.5.0) and we recalibrated the variant quality score of the jointly genotyped vcf with the GATK VariantRecalibrator. Next, we applied a hard filter with the criteria of QD < 2.0, FS > 60.0, MQ < 20.0, HaplotypeScore >13.0, MappingQualityRankSum < −12.5, or ReadPosRankSum < −8.0 on the recalibrated vcf as recommended in GATK’s best practice workflows. Finally, variants that passed the hard filter were annotated with an Ensemble Variant Effect Predictor [20].

### Identification of newly identified pleiotropic variants

By integrating a comprehensive human disease–SNP association database (VARIMED), Exome Aggregation Consortium (ExAC), and whole-exome sequencing (WES) of 50 brains from individuals with and without AD, we identified missense variants. In summary, we first selected 92 genes including APOE shared between the relevant genetic variants of CD and AD/D from VARIMED. Among those of genes, we selected 183 pathogenic genetic variants harbored in 58 genes. The pathogenicity of germline variants was determined based on the averaged functional impact of missense variants (mean score >0) from 16 algorithms, including SIFT [21] and Polyphen2 [22]. In Additional File 5, the list of 16 used algorithms is presented. We selected those 16 scoring methods based on the coverage (>90%) and the median score (range 0.2–0.3) criteria, and averaged their scores for each missense mutation. Among those 183 variants, we finally selected three variants in three genes as candidates for newly identified pleiotropic variants that might contribute to both CD and AD/D. Those three variants are (i) among the top 10 ranked pathogenic score (>0.7), (ii) not detected in individuals without AD in our WES samples, (iii) rare variants based on ExAC (allele frequency <1%) [23], and (iv) not reported in ClinVar, implying that they were not identified as mutations in human phenotypes [24].

### Validation of the pleiotropic variants for CD and AD/D

#### Molecular cloning of the selected variants and expression of the human protein, tau

Three of the selected germline mutations (*MTHFD1L* c.1688G>A; p.Arg563His, *DPP10* c.2254C>A; p.Gln752Lys, *ADIPOQ* c.268G>A; p.Gly90Ser) are reconfirmed in identical brain samples through Sanger sequencing. After the reconfirmation of germline mutations, full-length cDNA of human *MTHFD1L* (2937 bp, ENST00000367321.7) was chemically synthesized and mutant form *MTHFD1L* (c.1688G>A), *ADIPOQ* (735 bp, ENST00000320741.6; c.268G>A), and *DPP10* (2391 bp, ENST00000410059.5; c.2254C>A) were generated by mutagenesis. To append a 3×FLAG tag on either N-, C-terminal of wild-type and mutant form, we synthesized 3×FLAG DNA fragments by annealing oligos (Table 1).

**Table 1:** Oligos used to synthesize 3×FLAG DNA fragments.

	sense 5′–3′	anti-sense 5′–3′
(i) N-3×FLAG	ATGGACTACAAAGACCA	CTTGTCATCGTCATCCTTGTA
	TGACGGTGATTATAAAGAT	GTCGATGTCATGATCTTTATAA
	CATGACATCGACTACAAG	TCACCGTCATGGTCTT
	GATGACGATGACAAG	TGTAGTCCAT
(ii) 3×FLAG-C	GACTACAAAGACCATGACGG	CTACTTGTCATCGTCATC
	TGATTATAAAGATCATGACAT	CTTGTAGTCGATGTCATG
	CGACTACAAGGATGACGATG	ATCTTTATAATCACCGTC
	ACAAGTAG	ATGGTCTTTGTAGTC

Then, we performed overlap PCR with 3×FLAG and each cDNA as templates to make the final insert. We assembled each insert and linearized *Xho*1/*Eco*R1 double-digested form of pCIG2-C1 plasmid (modified from pCIG2) by using an EZ-fusion cloning kit (EZ016S, Enzynomics South Korea) to obtain pCIG-3×FLAG-h*MTHFD1L*, pCIG-3×FLAG-h*ADIPOQ*, and pCIG-3×FLAG-h*DPP10*. For an expression test, 3 μg of pCIG2-3×FLAG (Empty), pCIG2-3×FLAG-WT, and pCIG2-3×FLAG-mutant plasmid DNA were transiently expressed in Neuro-2a cell line (American type Culture Collection [ATCC], CCL-131) by using iN-fect reagent (15081, iNtRON South Korea) and cells were harvested after 72-h transfection. Neuro-2a is a fast-growing mouse neuroblastoma cell line and has been widely used to study Alzheimer’s disease [25]. Therefore, we used this cell line for the functional validation.

The expression levels of human proteins, such as tau (a known phenotype signature of AD), were estimated using western blotting. The cells were washed with phosphate-buffered saline (PBS) and lysed with 1× RIPA buffer (9806, Cell signaling Technology) in combination with protease inhibitor cocktail (P8465, Sigma USA). The lysates were cleared by centrifugation at 13,000 × *g* for 30 min and the protein concentration of the supernatant was determined using a Bradford protein assay (Bio-Rad). Total proteins were separated by NuPage 4–12% bis Tris–polyacrylamide gel electrophoresis in MES SDS running buffer (ThermoFisher Scientific), and then transferred to a PVDF membrane (GE Healthcare USA). The membranes were probed with specific antibodies. Immunocomplexes were detected using horseradish peroxidase-conjugated anti-rabbit, mouse antibodies followed by chemiluminescence detection (ECL, Amersham). The band intensities were quantified using NIH ImageJ software. We used specific primary antibody against CP13 (Anti-CP13, phospho-tau ser202) (39357, Cell Signaling Technology). Polyclonal tau (A0024) antibody was purchased from Dako, and PHF1 (phospho ser396/404) antibody was kindly provided by Dr. P. Davies. Anti-β-actin antibody was obtained from Sigma (A2228).

#### Analysis of the functional role of ADIPOQ in a cardiomyoblast cell (H9c2) and associated disease pathway

To evaluate the functional role of *ADIPOQ* on CD, we cloned *ADIPOQ* knockout (*ADIPOQ*-KO) cells using cardiomyoblasts (H9c2). Using a CRISPR-Cas9 system (px 330 all-in-one vector), we constructed *ADIPOQ*-KO cells [26], single-cell RNA-seq data of these *ADIPOQ*-KO cells were analyzed using the Chromium platform (10X Genomics). The single-cell RNA-seq reads were filtered based on the percentage of mitochondrial genes (<30%). All of doublet artifacts are removed using DoubletFinder [27]. From 15,423 cells and 800,233,426 reads of transcripts, we calculated the pseudobulk expression of all genes [28] to identify differentially expressed genes (DEGs) by comparing the reads with those from conventional RNA-seq of H9c2 cells (GSE89130). After quantile normalization between our pseudobulk expressions of genes and transcript abundance of H9c2, we selected DEGs using R software. The associated pathways and Gene Ontology (GO) of the selected DEGs were determined based on the *p* value of the gene set enrichment test [29] using the R software package clusterProfiler (https://git.bioconductor.org/packages/clusterProfiler).

#### Structural and functional differences of hearts and brains by the germline variants of ADIPOQ

We also examined anatomical and functional differences by the selected germline variant in *ADIPOQ*, such as volumes of heart, trends in numeric memory function, and asymmetricity of brains in a longitudinal manner from the UK Biobank. Of 502,591 individuals in the UK Biobank study, germline variants of 49,960 of individuals were sequenced using WES. Of these 49,960 of participants, we selected 310 who had a rare variant in *ADIPOQ* (ENST00000320741.6; c.268A), then selected 49,642 individuals who have a major allele in *ADIPOQ* (ENST00000320741.6; c.268G). To show the contribution of the rare variant of *ADIPOQ*, we selected 69 individuals from a minor allele group, and 275 from a major allele group based on a propensity score analysis [30], implying that other confounders such as age, level of obesity, and sex are similar between compared sets. The volume of hearts including the thickness of the heart wall, volume of ventricles, and aorta were estimated based on the cardiac magnetic resonance images (MRIs) using deep learning-based auto-segmentations [31]. Likewise, the longitudinal trends of numeric memory function of the individuals from the UK Biobank were also compared according to the selected allele groups of *ADIPOQ*.

## Results

### Overview of study

**Fig. 1:**
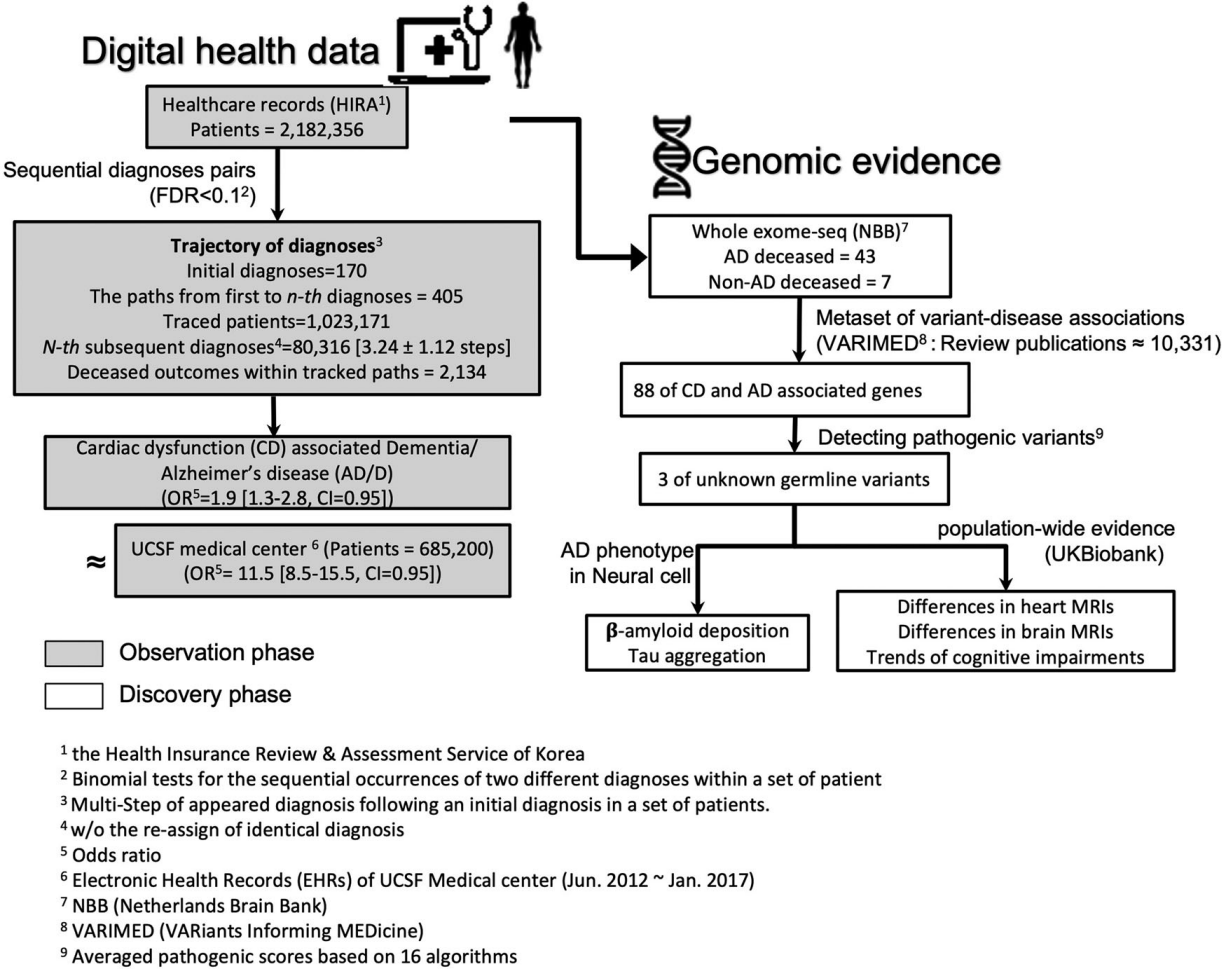
Identifying shared genetic architecture by repurposing scaled digital health data. Schematic workflow of a developing hypothesis in the observation phase based on digital health data (gray boxes) to study, using genomic data (white boxes). In the gray boxes, the database of HIRA and the electronic health records (EHR) of UCSF Medical Center were used to suggest the association of cardiac dysfunction (CD), such as hypertensive cardiac disease, with dementia and Alzheimer’s disease (AD/D); the public database of HIRA (Health Insurance Review and Assessment service) was used as a nationwide inpatient/outpatient diagnoses observation database in South Korea; the EHR of UCSF was used as the validation database. After presenting the propensity of CD amongst AD/D diagnoses, we examined the genetic associations between CD and AD/D in a discovery phase (i.e., white boxes).

The exploration and discovery phases of the present study are depicted in Fig. 1. The merged HIRA covered 55,291,171 diagnoses for 2,182,356 individuals with 43,545 death outcomes. The mean age of patients in the merged HIRA was 54.17 ± 23.26 years. We also utilized deidentified EMRs of UCSF Medical Center consisting of 685,200 patients for the validation of the identified diagnosis patterns from NISK of HIRA (Table 2).

**Table 2:** Summary of the datasets used.

	Datasets and features	Frequency
Observation phase	National inpatient/outpatient set of HIRA^a^	
	Total patients	**2,182,356**
	Selected patients^b^	**763,892**
	No. of men in the selected set	341,788 (44.7%)
	No. of women in the selected set	420,104 (55.2%)
	No. of diagnoses	**13,459,583**
	No. of unique diagnosis codes (ICD-10)^c^	5859 (5–3-letter level)
	Unique diagnosis high-level (3 letter)^d^	**1151**
	Mean of diagnoses age	54.19 (±23.26)
	Outcome of diagnoses	
	Deceased^e^	43,545 (5.7%)
	Alive^f^	1,252,114
	UCSF Medical Center^g^ (2012.01 to 2017.01)	
	Total patients	6,852,000
	Total cases of diagnoses	44,545,038
	No. of unique diagnoses codes (ICD-10-CM)	29,893
	Unique diagnosis codes with 3 letters	1831
	Mean of diagnoses age	48.67 (±23.41),
	Outcome of diagnoses	
	Deceased	2961
	Not deceased	143,996
	Pending	21,248,546
Discovery phase	VARIMED (VARiant Informing MEDicine)	
	No. of reviewed publication	**10,331**
	No. of SNPs (dbSNP IDs)	130,426 (129,890)
	No. of traits (disease/non-disease traits)^h^	4 223 (1 489/2374)
	No. of associations between SNPs and traits	**135,410**
	NBB (Netherlands Brain Bank)	
	Total patients (AD/non-AD)^i^	**50 (AD 43/non-AD 7)**
	Hippocampal formation (HF) samples	50
	Blood (BL) samples	50
	Mean deceased age (AD/non-AD deceased)	83.5±8/71.4±12.6
	Sex (AD deceased)	Male = 14; Female = 29
	Sex (non-AD deceased)	Male = 3; Female = 4
	Braak staging (AD/non-AD deceased)	5.02±1.1/0.71 ± 0.48
	UK Biobank	
	No. of total participants	502,543
	Participants with whole-exome seq (WES)^j^	49,960

^a^The Health Insurance Review and Assessment Service of Korea (HIRA). We utilized non-longitudinal sets consisting of randomly sampled in/outpatient sets built annually from 2009 to 2011 (www.hira.or.kr).

^b^To minimize the re-enrollment of patients into the 2011 set from the 2009 and 2010 sets, we selected only deceased patients from the 2009 and 2010 sets. In addition, we excluded records of non-disease-related diagnoses, including injuries, poisoning, and childbirth, using diagnosis codes.

^c^International Statistical Classification of Disease and Related Health Problems 10th Revision (ICD-10).

^d^Based on the hierarchical structure of ICD-10 codes, which consists of a 5-letter level for a disease with familial history and a 3-letter level for general disease classification, we used transformed diagnosis codes at the 3-digit level in this study.

^e^Detected outcomes in health insurance reviews (HIRA).

^f^Other non-deceased outcomes included ongoing patients, transferred, sent back, others, and discharged while alive.

^g^Deidentified electronic medical records (EMRs) from the University of California, San Francisco (UCSF) Medical Center (a tertiary-care university hospital).

^h^Counted based on MeSH terms (Medical Subject Headings, the National Library of Medicine’s controlled vocabulary) for traits including eye color and diseases such as asthma.

^i^All collected samples were of Western European ancestry.

^j^We analyzed participants with WES data to validate the phenotypic effect of the germline variant of interest.

Bold characters emphasize the numbers of the table.

We traced the temporal relationships between diseases diagnosed within individuals to investigate the possibility of a pleiotropic effect on genetic risk factors. To systematically trace the disease diagnosis trajectories, we simplified the timeline for each patient by recording the first instance of each primary diagnosis, such that any later diagnosis of the same disease was removed. We used our previous approach, directed acyclic graph (DAG) modeling approach to recapitulate the pattern of disease diagnoses by time [32]. In DAG modeling, the trajectory consists of the first node for a set of patients diagnosed with a disease *i*, and nodes are connected via directed acyclic edge presenting subsequent diagnoses occurred frequently than random. The details of our DAG-based approach were published previously [6, 18]. The concept of our DAG-based approach is presented graphically in Additional file 2. The significance of sequential patterns of diagnoses and directionalities were determined by the *p*-value of the binomial test (FDR < 0.1). In our model, there were 405 disease trajectories traced in total, and our model showed 3.24 steps of disease diagnoses after the initial diagnosis on average (Table 3). After excluding co-occurring diagnoses, which may be associated with the milieu of the 405 disease trajectories, we selected a diagnosis pattern starting from CD to AD/D to examine a possible pleiotropic effect between the diseases through shared genetic risk factors.

**Table 3:** Statistics of diagnosis trajectory analysis using the set of HIRA.

	Features	Frequency
Trajectory^a^ = the first and followed nodes linked via edges	No. of trajectories	**405**
Node = Diagnosed patients as a *disease i*	Total type of diagnosis	604
	The 1st diagnosis	**170**
	Patients in the 1st diagnosis	**1,023,171**
	Fatal outcomes of diagnosis	2 134
Edge (Directed acyclic edge) = Sequence between *diagnosis i* and *j* (FDR < 0.1)^b^	Subsequent diagnoses after the 1st diagnosis	**80,316**
	Mean of steps diagnoses in a trajectory	**3.24** ± **1.12**

^a^The trajectory consists of the first node, representing a set of patients diagnosed with disease *i*, succeeding nodes are connected via a directed acyclic edge, representing subsequent diagnoses that occur more frequently than randomly.

^b^All the presented edges were statistically significant (Relative association (RA) for the co-occurrence of diagnosis *i* and *j* > 1 and FDR of the binomial test for the co-diagnosis of diagnosis *i* and *j* < 0.1; FDR of the binomial test for the sequential directionality of diagnosis dates <0.1). The details of the diagnosis trajectory model are presented on the following website (https://www.youtube.com/watch?v=jJMds31-e2g).

Bold characters to emphasize the numbers of the table.

After we validated a sequential pattern of disease diagnoses, including CD and AD/D, based on the HIRA and UCSF datasets, the shared genetic risk contributions (i.e., a pleiotropic effect) were examined using population-wide evidence from the UK Biobank data and experimental validations.

### Validation of the association between CD and AD/D

The 405 disease trajectories modeled based on the HIRA dataset are presented in Additional file 3. Our DAG model demonstrates the real-world phenomena, including the known clinical burden of hospital deaths, and regional pattern of diagnoses prevalence. For example, chronic obstructive pulmonary disease (COPD) patterns in patients with dementia and the elderly were captured in our model with 854 fatal outcomes via the sequential diagnoses including pneumonia, which reinforces the findings of previous studies (Additional file 2) [33, 34].

**Fig. 2:**
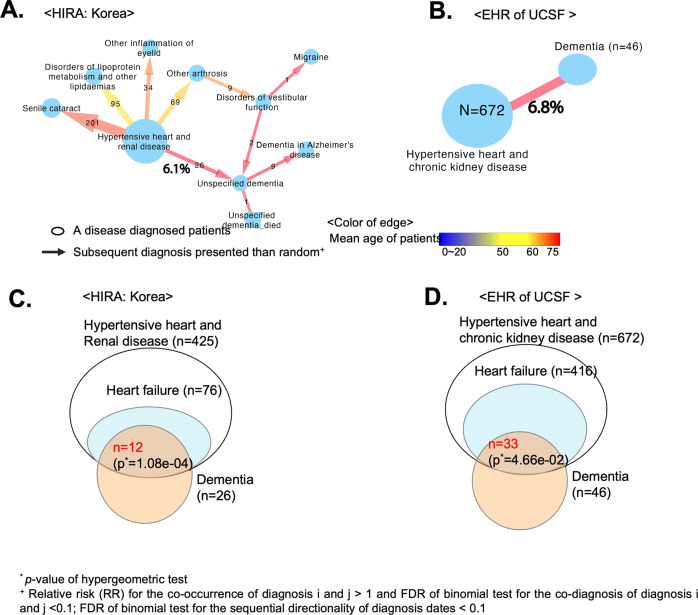
The preceding diagnosis of heart disease before dementia. **A**, **B** Traced models of disease diagnosis patterns using the directed acyclic graph (DAG) model. Our DAG model consisted of a node for the disease diagnosis and an edge for subsequent diagnoses presented that were not random (FDR < 0.1). **A** From the HIRA dataset, we found that 6.1% of the patients suffering from hypertensive heart and renal disease (*n* = 425) were diagnosed with unspecified dementia and Alzheimer’s disease (a major subtype of dementia). **B** We validated whether the identical pattern was repeated in the independent dataset obtained from the electronic health records (EHR) of the UCSF Medical Center. Of the 672 patients with hypertensive heart and kidney disease, 6.8% (*n* = 46) were diagnosed with dementia. **C**, **D** Due to the vague definition of the “hypertensive heart and kidney disease” diagnosis, we looked into a more detailed diagnosis code at the 5-digit level of ICD-10. **C** In the HIRA dataset, approximately 46% of dementia diagnoses made in patients who already had hypertensive heart and renal diseases were identified after heart failure. Therefore, the diagnosis of dementia after the diagnosis of hypertensive heart and renal disease was significantly enriched by cardiac disease (CD) (*p* = 1.08E–04, hypergeometry test). **D** Likewise, the EHR from the UCSF Medical Center also showed an identical pattern. Of the 46 dementia diagnoses made in patients with pre-existing hypertensive heart and renal disease, 33 were identified after the diagnosis of heart failure. The diagnosis of dementia after the diagnosis of hypertensive heart and kidney disease was enriched by cardiac diseases, such as heart failure (*p* = 4.66E–02, hypergeometric test).

The CD to AD/D diagnostic path identified in patients from South Korea is shown in Fig. 2A. In total, 425 patients were diagnosed as having hypertensive heart and renal disease, and then presented diverse disease status including aging-related diseases (senile cataracts) and known comorbidity of cardiovascular diseases (hyperlipidemia) [35] within 1 year. Of those 425 patients, 6.8% (*n* = 26) were diagnosed with AD/D. Patients who were diagnosed with dementia following hypertensive heart and kidney disease were enriched with those having hypertensive heart disease (*p* = 1.08E–04, hypergeometric test; Fig. 2C). We note that diagnoses of vascular dementia were not included among these 26 patients. Although there is a growing number of reports for the coexistence of heart failure and dementia, the shared risk factors are unclear [36] and warrant further analysis.

To examine confounders for this propensity for nonvascular AD/D among patients with hypertensive heart failure, we scrutinized independent medical records. We hypothesized that repeated representation of co-occurring diagnoses from an independent dataset shows shared genetic risk factors between diseases. Indeed, the same pattern of diagnoses is observed in the EMR of UCSF Medical Center (Fig. 2B). The UCSF Medical Center EMR provided deidentified data from 816504 patients from 2011 to 2018. In summary, among 672 patients with hypertensive heart and kidney disease, mostly those with hypertensive heart failure (*p* = 4.66E–02, hypergeometric test; Fig. 2C), 6.8% (*n* = 46) were also diagnosed with dementia. HIRA and UCSF Medical Center EMR were gathered for nonbiomedical research purposes, indicating a possibility of diagnoses primarily for the billing of health insurance. However, for confirmation of CD and AD/D, general guidelines from the American Heart Association (AHA) [37] and the *Diagnostic and Statistical Manual of Mental Disorders: 5th Edition* (DSM-5) [38] require evidence-based evaluation of patients, such as MRIs and Mini-Mental State Examination (MMSE) score. Therefore, we were confident of the categorization of the disease patients and the sequential pattern of their diseases. Moving forward to the discovery phase of our study, we sought to verify the shared underlying mechanisms in disease trajectories.

### Identification of pleiotropic variants contributing to AD/D and CD

We analyzed WES of 50 samples from deceased individuals consisting of 43 patients with AD and seven individuals from normal group. Since we were mainly interested in the genetic risk of AD/D and CD in the elderly (Fig. 2), we included only the group of subjects with AD are those with late-onset cases (age of diagnosis ≥65, mean deceased age 83.5 ± 8 years). A summary of the demography of patients whose samples were analyzed by WES is shown in Table 4.

**Table 4:** Candidates with pleiotropic features (i.e., shared genetic risks) between CD and AD/D.

Genomic location^a^	Gene symbol	Ref/Alt2^b^	Average of pathogenic score^c^	No. of AD^d^	Allele frequency		Clinical significance (ClinVar)
					ExAC (all)	ExAC (Finnish cohort)	
Chr6, 150949095	*MTHFD1L*	G/A	0.815	3	0.01264	0.0065	NS
Chr2, 115840821	*DPP10*	C/A	0.792	2	0.00308	0.00076	NS
Chr3, 186854237	*ADIPOQ*	G/A	0.861	1	0.00302	0.00045	NS

None of the selected variants were detected in the non-AD samples.

*NS* not significant.

^a^Genomic assembly version of GRCh 38.

^b^Reference nucleotides (Ref) and alternative nucleotides (Alt).

^c^Average pathogenic scores from 16 algorithms (SIFT, Polyphen2_HDIV, Polyphen2_HVAR, MutationTester, PROVEAN, REVEL, CADD, fathmm-MKL, Eigen-PC-raw, GERP++, PolyP100way_vertebrate, PolyP20way_Mammalian, phastCons100way_vertebrate, phastCons20way_mammalian, VEST3, and Siphy_29way_logOdds).

^d^Number of patientswith Alzheimer’s disease (AD) in our NBB WES dataset (43 AD and seven non-ADsamples).

**Fig. 3:**
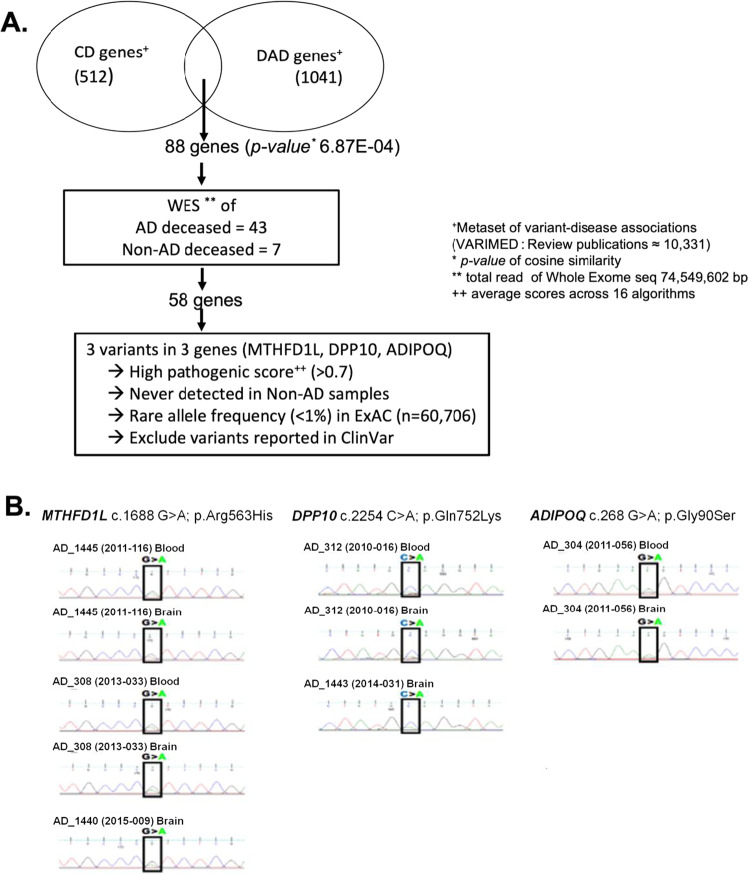
Identification of the candidates of the pleiotropic variants for CD and AD/D. **A** Integration of the variant–disease associations metaset from our VARIMED and the whole-exome sequencing (WES) dataset. We selected 3 variants in *MTHFD1L*, *DPP10*, and *ADIPOQ* for further validation. **B** Reconfirmation of selected germline variants using Sanger sequencing.

We analyzed 1,327,403 bp of exonic regions of 88 genes of CD- and AD/D-associated genes (Fig. 3A). Those 88 genes were selected from our comprehensive human disease–SNP association database (VARIMED), a manually curated database of disease–SNP associations, containing over 100 features of association studies from 8962 human genetics papers covering 2376 diseases and traits [9]. The identified set of 88 genes commonly associated with CD and AD/D is shown in Additional file 4. For example, it is known that rare variants of *APOB* elevate the level of low-density lipoprotein cholesterol (LDL-C) and then ultimately increase the risk of the AD [39]. Meanwhile, genetic mutations of *APOB* are also associated with the risk of coronary artery diseases and CD [40, 41]. Similarly, other notorious shared genetic risks, such as variants of lipoprotein lipase (LPL), shared between CD and AD/D were detected [42, 43]. In addition, variants with lower pathogenicity score including one variant in ADIPOQ (score = 0.227) were excluded from further analysis (Additional file 4). We filtered 184 germline variants of 58 genes as shared genetic risks of CD and AD/D, and then finally selected three variants in three genes (*MTHFD1L*, *DPP10*, *ADIPOQ*) as candidates with pleiotropic features for the comorbidity of CD and AD/D (Additional file 4). Those three variants were selected based on the newly identified and rare variants in ExAC (<1%) and ClinVar [24], and the averaged pathogenicity scores (>0.7; range [0,1]) from 16 algorithms including SIFT [21] and Polyphen [22], which quantify the functional impact of missense variants (Fig. 3A). The overall distribution of pathogenicity scores across the 16 methods we used are presented in Additional file 5. The three variants were detected in six samples from patients with AD, but in no samples from those without. The average of pathogenicity scores of the variants are presented in Table 4. The variant of *MTHFD1L* was detected in samples from 3 patients with AD in our NBB WES data (c.1688G>A; p.Arg563His) with pathogenicity score of 0.81, and rarely detected in the ExAC database covering over 60706 individuals. Similarity, variants of *DPP10* and *ADIPOQ* were detected in two and one of our AD samples with high pathogenic scores (0.79 and 0.86, respectively), and rarely detected in the ExAC database. Therefore, we selected those variants as newly identified candidates, which may be associated with CD and AD/D.

Because our WES data were generated from brain samples of patients with AD, we confirmed the variants of the matched blood samples experimentally, if possible, to exclude the possibility of somatic mutations. Three selected germline mutations (*MTHFD1L* c.1688G>A; p.Arg563His, *DPP10* c.2254C>A; p.Gln752Lys, *ADIPOQ* c.268G>A; p.Gly90Ser) were reconfirmed in brain samples and available blood samples from identical individuals through Sanger sequencing (Fig. 3B).

The rare variants *MTHFD1L* (c.1688G>A), *DPP10* (c.2254C>A), and *ADIPOQ* (c.268G>A) were selected as candidates for pleiotropic variants for CD and AD/D.

### Functional validation of the pleiotropic variants using in vitro experiments

**Fig. 4:**
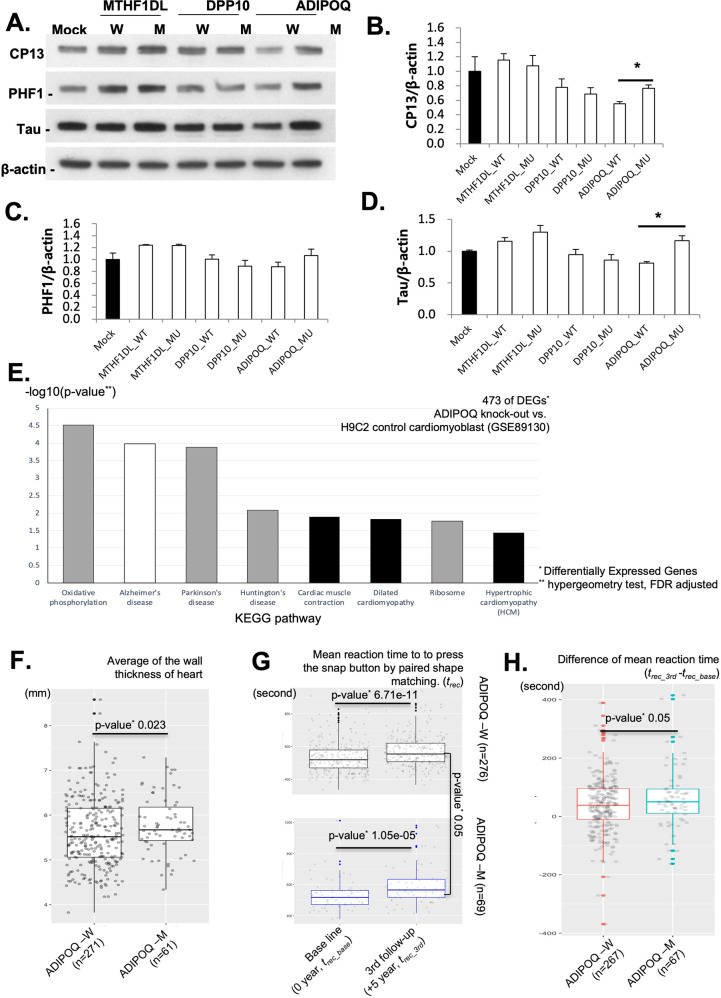
Validation of pleiotropic effects for CD and AD/D. **A–D** Validation of the functional impact of the 3 identified AD/D variants. We constructed site-directed mutant Neuro-2a cell lines (*MTHFD1L*-M, *DPP10*-M, and *ADIPOQ*-M). We assessed the abundance of each cell line of CP12, PHF1, and tau proteins via three biological replicates. **A** Loading abundance of CP13, PHF1, and tau proteins, as well as β-actin in neuronal cell lines by transfected genes. ‘M’ = mutated. ‘W’ = wild type. **B**–**D** Levels of aggregation of CP13, PHF1, and tau normalized by β-actin. *ADIPOQ*-M displayed abnormal aggregation of tau and CP13 (D) (*p* < 0.05, *t*-test). **E** Analysis of an enriched pathway of 473 selected differentially expressed genes (DEGs) (FDR *p* < 0.05, log_2_ fold change >5) in *ADIPOQ* knockout cells, H9c2 (rat cardiac cell). Cardiac dysfunction and cognition impairment pathways were enriched in 473 DEGs (FDR *p* < 0.05, hypergeometric test). **F–H** Functional impact of the *ADIPOQ* variant on a population scale using the UK Biobank. We selected 69 individuals from the minor allele group of *ADIPOQ* (c.268G>A) and 276 from the major allele group based on the propensity score matching analysis. **F** The average heart wall thickness across 16 sites was significantly increased in the *ADIPOQ*-M group (*ADIPOQ* c.268A) (*p* = 0.0023, Wilcoxon test). **G** Differences in mean reaction time (RT), calculated over 12 rounds, to press a “snap” button when both cards presented matched correctly. The *X*-axis represents the difference in the measured RTs between the initial assessment (0 years) and the third assessment (5–10 years later). Each plot shows the RTs by allele group (*ADIPOQ*-W and *ADIPOQ*-M). **H** Difference in the mean RTs between the baseline and the third assessment. The minor allele group (*ADIPOQ*-M) showed a longer mean RT than the major allele group in the third assessment compared to the first assessment (*p* < 0.05, t-test).

The degree of abundance of CP13 (phosphorylated tau Ser202), PHF1 (phosphorylated tau Ser396/404), and tau proteins in the neuronal cell lines by transfected genes is shown in Fig. 4A. We assessed the abundance of CP12, PHF1, and tau proteins for three biological replicates for each cell line. The abnormal aggregation of CP13, PHF1, and tau is a well-known neuropathology of AD/D [44–46]. We quantified the aggregation of CP13, PHF1, and tau across the status of genetic variants that we selected (three repeats per condition). Although the variants of *MTHFD1L* (*MTHFD1L*-M) and *DPP10* (*DPP10*-M) showed similar levels of expression for CP13, PHF1, and tau, the site-directed mutant *ADIPOQ* (*ADIPOQ*-M; *ADIPOQ* c.268G>A) displayed abnormal aggregation of tau and CP13 (Fig. 4B–D; *p* < 0.05, *t-*test). Therefore, the identified *ADIPOQ* variant (c.268G>A) seems to contribute the aggregation of tau and CP13 (phosphorylated tau Ser202).

Owing to the poor transfection efficacy of ADIPOQ-M (*ADIPOQ* c.268G>A) in a cardiac cell line (H9c2), we constructed an *ADIPOQ* knockout (*ADIPOQ*-KO) cardiac cell using a CRISPR-Cas9 system (px 330 all-in-one vector, Additional file 6). Then, we constructed the pseudobulk expression of *ADIPOQ*-KO across 14116 cells by aggregating the count matrix of single-cell RNA-seq. The pseudobulk expression of 15791 genes was calculated based on aggregating the raw read count as depicted in the Methods section and a previous attempt [28]. By comparing the RNA-seq result from the Gene Expression Omnibus (https://www.ncbi.nlm.nih.gov/geo/, accession number: GSE89130), 473 DEGs in *ADIPOQ*-KO were determined (FDR *p* < 0.05 and log_2_ of fold change >5). We conducted a quantile normalization followed by a comparison of the pseudobulk expression to a cardiomyoblast control (GSE89130, bulk RNA-seq results). Here, we note that the identified variant (*ADIPOQ* 268G>A) is a missense variant with a high pathogenicity score (0.86, Additional File 4), meaning the protein of the *ADIPOQ* variant would be damaging or truncated. Therefore, we examined the overall impact of DEGs of *ADIPOQ-KO* to prioritize associated biological function for further validation. The overall gene expression between *ADIPOQ-KO* and GSE89130 was similar (Pearson correlation coefficient 0.721, *p* = 1.54E–125) indicating that the batch variation by sequencing technology was negligible (Additional file 6). The 473 DEGs are enriched with CD and cognition impairment pathways including AD, cardiac muscle contraction, and hypertrophic cardiomyopathy (FDR *p* < 0.05, hypergeometry test; Fig. 4E). The enriched gene ontology (GO) terms of those DEGs show favorable support such as *sarcomere* (GO:0030017; FDR *p* = 0.0049), *muscle system process* (GO:0003012, FDR *p* = 1.23E–05), and *contractile fiber* (GO:0043292, FDR *p* = 0.00049, Additional file 7). The functional impact of *ADIPOQ* deletion shows that the alteration of overall gene expression is associated with cardiac function, including hypertrophic cardiomyopathy.

In summary, we confirmed that the variant of *ADIPOQ* (c.268G>A) contributes to abnormal aggregation of tau, and that gene expression altered by the knockout of *ADIPOQ* is peculiarly associated with dysfunction of cardiac muscle.

### Functional validation of the pleiotropic variants using population-wide data

We scrutinized phenotypic differences by the germline variant of *ADIPOQ* (*ADIPOQ* c.268G>A). Of 502,591 individuals in the UK Biobank, 49,960 participants have a matched WES data (Table 2). Of them, we selected 310 who have the rare variant in *ADIPOQ* (ENST00000320741.6; c.268A), then selected 49,642 individuals who have the major allele in *ADIPOQ* (ENST00000320741.6; c.268G). We selected 69 individuals from the minor allele group, and 276 individuals in the major allele group based on the propensity score matching analysis [47], thereby minimizing differences in other confounders, such as age, level of obesity (measured by the body mass index), and sex, between the compared sets (Additional file 8).

The volume of hearts, including the thickness of heart wall, volume of ventricles, and aorta, were estimated based on cardiac magnetic resonance images (MRIs) using deep learning-based auto segmentation [31]. The average of the wall thickness of the heart across 16 sites is significantly increased in the *ADIPOQ*-M group (*ADIPOQ* c.268A, *p* = 0.0023 in one-sided Wilcoxon test for whether the thickness of *ADIPOQ*-M group is greater than W); mean of the wall thickness in *ADIPOQ*-W = 5.60 ± 0.76 mm; mean of the wall thickness in *ADIPOQ*-M = 5.77 ± 0.64 mm (Fig. 4F). The wall thickness measured from 16 different sites in the heart between *ADIPOQ*-W (major genotype GG) and *ADIPOQ*-M (minor genotype AG) is shown in Additional file 9. However, other screened phenotypes from heart MRIs, such as ventricular volume and atrial volume, are similar in the allele group of *ADIPOQ*, indicating that a thick heart wall associated with the germline variant of *ADIPOQ* (*ADIPOQ* c.268G>A) is independent of heart volume.

We also examined the genetic contribution of the *ADIPOQ* variant (*ADIPOQ* c.268G>A) for AD/D. Based on the reliability of the cognitive tests in the UK Biobank [48], we selected mean reaction time (RT) of participants. Within an assessment, the RT was measured for 12 rounds by pressing a snap button for which both cards presented matched correctly. We compared the mean RTs between the results of the initial assessment (0 year) and the third assessment (5–10 years later). We omitted the second assessment due to the lack of assessment results. The longitudinal trends of cognition and processing functions are decreased in both *ADIPOQ* allele groups with the participants’ aging (Fig. 4G). The mean age of all participants was 53.95 years (*ADIPOQ*-W) and 53.45 years (*ADIPOQ*-M) in the initial assessment, respectively (Additional file 8). Meanwhile, the mean RTs between *ADIPOQ*-W and *ADIPOQ*-M were found to be similar from the initial assessment *(ADIPOQ*-W = 531.94 s; *ADIPOQ*-M = 531.60 s, *p* = 0.4, *t*-test), however the minor allele group (*ADIPOQ*-M) showed a significant increase at the third assessment (*ADIPOQ*-W = 569.87 s; *ADIPOQ*-M = 596.92 s, *p* < 0.05, *t*-test). Similarly, the difference of the mean RT alterations between allele groups is shown in Fig. 4H. The *Y*-axis of Fig. 4H indicates the difference between the mean RTs at the baseline and third assessment; the larger value of *Y*-axis of Fig. 4H indicates degeneration of the cognitive processes and the slowing of RT of participants after the first assessment. At the third assessment time, the minor allele group *ADIPOQ* (*ADIPOQ*-M, *ADIPOQ* c.268G>A, Minor AG) showed an almost 155.24% longer mean RT after the first assessment than the major allele group (mean difference of the RT between the first and third test; major GG = 39.16 ± 95.7 s, minor AG = 60.70 ± 108.44 s, *p* < 0.05, *t*-test; Fig. 4H). Although the onset of AD/D was undetected from the health care records of these individuals owing to their relatively young age (mid-50s), our examination shows the contribution of the germline variants of *ADIPOQ* (c.268G>A) for the phenotype of AD/D, such as degeneration of the processing and response.

These results indicated that we successfully validate potential pleiotropic effects of the variant of *ADIPOQ* (c.268G>A) for the phenotype of AD/D (aggregation of tau, and the longitudinal trend of cognitive degeneration; *p* < 0.05), and the phenotype of CD (thickened muscle in the wall of the heart; *p* < 0.05) (Fig. 4A–H). The repeated patterns of paired diagnoses (i.e., CD and AD/D) (Figs. 1–3) from the population-scale EMRs are based on the pleiotropy of *ADIPOQ*. Our identified germline variant of *ADIPOQ* (c.268G>A) is a genetic risk shared between CD and AD/D.

## Discussion

In the present study, we hypothesized that a repeated diagnosis of two diseases within a single patient is associated with a shared biological mechanism, such as a pleiotropic variant, and can be validated via big data. Using the systematic approach of our previous work (i.e., DAG modeling) [6], we observed from the NISK of the HIRA and UCSF data that a CD diagnosis, such as hypertensive heart disease or heart failure, is particularly frequent amongst patients with AD/D (odds ratio 11.5 [8.5–15.5, 95% confidence interval (CI)]). We identified three missense variants, including *ADIPOQ*, which might have a pleiotropic role in both diseases. Functional evidence covering tau aggregation, transcriptional impact of *ADIPOQ* knockout for cardiomyoblasts, and the quantitative assessment of cognition and cardiac muscle structures persistently indicate the pleiotropy of the *ADIPOQ* variant (c.268G>A) in CD and AD/D.

Although the expression *ADIPOQ* has been mainly observed in fat tissue, genetic variants of *ADIPOQ* showed association with diverse disease traits, such as heart failure and dementia. For example, the release of adiponectin, encoded by *ADIPOQ*, in epicardial adipose tissues contributes to the defense mechanism for myocardial oxidative stress [49]. Moreover, chronic adiponectin deficiency causes cerebral insulin resistance, leading to AD-like cognition impairments and Aβ deposition in aged mice [50]. Functional contributions of *ADIPOQ* for both of CD and AD/D been suggested previously, whereas confirmation of pleiotropic loci of *ADIPOQ* has hitherto been lacking.

Although a plethora of GWAS studies shed light on the genetic risks shared between diseases and traits, they are mainly based on statistical support from a population, calling for independent functional validation. However, analysis of multifunctional genes like that for *ADIPOQ*, which is involved in regulating fat metabolism and obesity-associated cognitive decline [51], requires multiple evidences for identification of their functional roles. Here, the power of big data analytics, including next-generation sequencing analysis and our disease trajectory modeling, catalyze the identification and validation of newly identified pleiotropic variants for CD and AD/D and showed that significant genetic evidences is indispensable to interpret data-driven analysis in medicine. Therefore, investigation of national projects to exploit the human genome aggregating phenotype data, such as AllofUs (https://allofus.nih.gov/) and FinnGen (https://www.finngen.fi/en), would accelerate the identification of genetic architecture underlying human diseases.

The present study has several limitations. Although we validated phenotypic impact for cardiac muscle alongside the variant of *ADIPOQ* (c.268G>A) using the UK Biobank, underlying transcriptional evidence remained unclear because of the poor transfection efficacy of *ADIPOQ* (c.268G>A) in H9c2 cells. The suggested pleiotropic locus of *ADIPOQ* shows functional impact for CD- and AD/D-asociated phenotypes (i.e., tau aggregation and hypertrophic cardiac muscle). The chronologic order of these diseases (CD followed by AD/D) and late-onset propensities are open questions. We also acknowledge that the suggested pleiotropic locus of *ADIPOQ* (c.268G>A) is a rare variant (minor allele frequency [MAF] <1% in ExAC), whereas the known incidence of CD and AD/D is substantial. While it is possible that common variants (MAF > 5%) are also associated with CD and AD/D, the functional impact of rare variants has been shown to have more substantial contributions [52]. The influence of confounders, including aging, as well as the subtle effects of common variants, require further study. Therefore, the presented Additional file 4 would pave the way to identify additional pleiotropic loci of CD and AD/D by conjugating genomic data and linked phenotype data, such as that in the UK Biobank, AllofUs, and FinnGen.

## Supplementary information

Supplementary Methods

Supplementary Figure 1

Supplementary Data 1

Supplementary Data 2

Supplementary Figure 2

Supplementary Figure 3

Supplementary Data 3

Supplementary Table 1

Supplementary Figure 4
