# Supplementary material for: Identification of a pleiotropic effect of ADIPOQ on cardiac dysfunction and Alzheimer’s disease based on genetic evidence and health care records
Source: Transl Psychiatry. 2022 Sep 16;12:389. doi: 10.1038/s41398-022-02144-0 (PMC9481623; doi:10.1038/s41398-022-02144-0)
Supplement: Supplementary file 1 — Supplementary Methods [file 41398_2022_2144_MOESM1_ESM.docx]

**Identification of a pleiotropic effect of *ADIPOQ* on cardiac dysfunction and Alzheimer disease based on genetic evidence and** **health care records at scale**

**Additional File 1. Supplemental Methods**

Population-wide administrative healthcare records

*Data of National Inpatient Set in Korea*

Data set used in this analysis was from National Inpatients Set of Korea from Health Insurance Review & Assessment Service (HIRA; www.hira.or.kr). HIRA involves 13% of random sampled hospitalized patients in all Korean hospital for each given year. Out of sampled hospitalized patients, HIRA data covers all primary and secondary diagnose records for all types of hospital visit within a year. HIRA set presented unique identifiers of each patient and assigned diagnose records with exact date, but associated admission-discharge date records were not involved. All of diagnose records were coded using ICD-10. HIRA set have been collected per each year without unique patient identifier across data generation years, and we used merged HIRA data involving 3 sets for every year from 2009 to 2011. In order to prevent the redundancy of merged set, we only used records of deceased individuals and their 1-year diagnose records in 2009 and 2010 data of HIRA, and integrated with the latest version generated in 2011. Overall, HIRA covers 55,291,171 of diagnose records, which were assigned for 2,182,356 patients in each three years, 2009, 2010 and 2011, in Korea. Among them, we selected over 13M of year round diagnosis records including inpatient and outpatient cases for 763,892 of hospitalized individuals.

Utilized diagnose code systems in HIRA, ICD-10 system has a hierarchical structure, where codes can be rounded to a general parent diagnosis code. We used this structure to round all codes to level 3 codes.

Significance of disease comorbidity with time orders

Based on a previous attempt, we used relative association (RA) to quantify the co-occurrences of disease pair (*Disease* *i*-*Disease j*) within 1-year [1]. RA measures relative ratio of disease co-occurrences over incidences of compared disease pair. The starting point of our RA measure is the database (HIRA) containing the diagnoses that *C_ij_* led to all hospitalization (*N*) of each disease identified by an ICD-10 code. We denote the incidence of disease *i* with *Ii*, and the number of patients who were diagnosed with diseases *i* and *j* with *C_ij_*. The co-occurrence value, RA, between diseases can be quantified via, RA=*C_ij_*/*C_ij_*^*^, where *C_ij_*^*^=*I_i_*• *I_j_*/*N* is the random expectation value of *C_ij_*. The statistical significance of disease comorbidity were determined using binomial test based on previous study [2]. After calculating RA values over all pairs of disease, we selected disease co-occurred pairs with RA>1 and FDR adjusted p-value of binomial test less than 0.1 for each country.

We also quantified directionality of disease co-occurrences (*Disease i* 🡪 *Disease j*) using date of diagnose or associated admission date. We define the directionality *δ*_i🡪j_ of the link connecting disease i to disease j as:

$\delta_{i\to j}=\frac{\sum_{p=1, p\in C_{ij}}^{{|C}_{ij}|} \sin(d_{jp}-d_{ip})}{|C_{ij}|}$ [1].

Where *d_jp_* indicates date of admission (or diagnose) of *p-th* patient for *j-th* disease, and *C_ij_* means set of common patients diagnosed as *i-th* and *j-th* disease. A value of *δ*_i🡪j_ > 0 indicates that mean date of admission (or diagnose) by i-th disease is earlier than j-th disease, whereas *δ*_i🡪j_ < 0 denotes reverse case. To reduce redundancy and meaningless associations of disease co-occurrences and directionality, multiple diagnose or re-visit with same disease cases were removed and only utilized initial date of diagnose (or month of admission and assigned diagnosis). The statistical significance of *δ* was determined by binomial test as conducted in previous study (FDR adjusted p-value <0.1) [2]. Finally, using HIRA set, we utilized selected pairs of disease co-occurrences (RA >1, FDR <0.1) having directional order of onset (*δ*_i🡪j_ ≠0, FDR <0.1) in further analysis.

Trajectory of diseases and deaths

We constructed time-lines between disease co-occurrences by transferred patients as following identified disease onset pairs and orders. The trajectory of diseases (i.e. ordered disease onsets) consisted of the patient had the diagnose record assigned in the order specified diseases. In previous, we determined order of disease onset regarding statistical significances of disease co-occurrences (RA>1 and FDR < 0.1) and time orders (*δ*_i🡪j_ ≠0, FDR <0.1) for all possible disease pair. Based on the transferred patients between two pairs of ordered disease onset, we organized multiple steps of disease-to-disease trajectories by merging two of diseases-disease into three steps of diseases (Disease 1🡪 2🡪3). For example, a path of diagnose order consisting three steps of disease onset was identified by combining pairs with overlapping diagnoses by sharing patients (Disease 1🡪Disease 2 and Disease 2🡪Disease 3 combined to Disease 1🡪Disease 2🡪Disease 3). They were subsequently extended with more overlapping pairs to obtain even longer trajectories. A greedy approach was used to find further step of disease paths covering the most patients. The pairs of diseases were sorted in descending order according to their common patient count. Pairs with an overlapping diagnosis were found starting from the top of the list and the number of patients following the full trajectory was counted. We stopped when the trajectories had no patients following them. Then, time-resolved frame of diseases onset was visualized as directed graph consisting of nodes for diseases and directed edges for ordered co-occurrences.

In case of HIRA, we filtered disease-to-disease timelines with meaningless orders of diseases, such as common influenza infections after chronic hypertension diagnoses. Using association rule mining[3], we identified frequently co-occurred diseases by group of disease timelines, which were determined by initial diseases (e.g. disease timelines with disease X in first step). Then, we selected differentially co-occurred disease for each group of disease timelines by normalizing overall specificity of selected diseases. For example, by association rule mining (Apriori algorithm), “angina pectoris” and “bronchitis” were selected as frequently co-occurred diseases among disease timelines started with “acute myocardial infarction”. In other sets of disease time lines with various initial disease states, two of them pick “angina pectoris” as co-occurred disease, whereas “bronchitis” co-occurred with 100 sets of disease. Based on these overall association numbers, we determined degree of specificity of “angina pectoris” for “acute myocardial infarction” as 0.5, 1/(No of associated disease sets). We determine differential associations between diseases in a set of diseases timelines by third quantile values in calculated degree of specificity (1/(No of associated disease sets) > 3rd quantile). Finally, out of disease time line start with “acute myocardial infarction”, a timeline with “angina pectoris” was selected by differential association rule mining, but a timeline with “bronchitis” was removed for further analysis.

All after building of ordered disease (or diagnose) co-occurrence paths, we merged associated outcomes of patients (Disease j - died with disease j). We note that died outcome for each patient’s disease diagnoses was presented without causal relationships. Thus, in constructed disease trajectories, outcome of each disease onsets were presented without directionality. In addition, time intervals between a disease onset and an outcome were absences in our datasets.

**Code availability**

Source code was deposited in https://github.com/hypaik/HCUPSIDCA_trajectory_tracking

**References**

1. Park J, Lee DS, Christakis NA, Barabasi AL. The impact of cellular networks on disease comorbidity. Mol Syst Biol [Internet]. 2009/04/10. 2009;5:262. Available from: http://www.ncbi.nlm.nih.gov/entrez/query.fcgi?cmd=Retrieve&db=PubMed&dopt=Citation&list_uids=19357641

2. Jensen AB, Moseley PL, Oprea TI, Ellesøe SG, Eriksson R, Schmock H, et al. Temporal disease trajectories condensed from population-wide registry data covering 6.2 million patients. Nat Commun [Internet]. 2014 [cited 2015 Jan 29];5:4022. Available from: http://www.pubmedcentral.nih.gov/articlerender.fcgi?artid=4090719&tool=pmcentrez&rendertype=abstract

3. Agrawal R, Mannila H, Srikant R, Toivonen H, Verkamo AI. Fast discovery of association rules. American Association for Artificial Intelligence; 1996 [cited 2015 Sep 16];307–28. Available from: http://dl.acm.org/citation.cfm?id=257938.257975
