# Supplementary material for: Identification of a pleiotropic effect of ADIPOQ on cardiac dysfunction and Alzheimer’s disease based on genetic evidence and health care records
Source: Transl Psychiatry. 2022 Sep 16;12:389. doi: 10.1038/s41398-022-02144-0 (PMC9481623; doi:10.1038/s41398-022-02144-0)
Supplement: Supplementary file 2 — Supplementary Figure 1 [file 41398_2022_2144_MOESM2_ESM.pptx]

## Slide 1
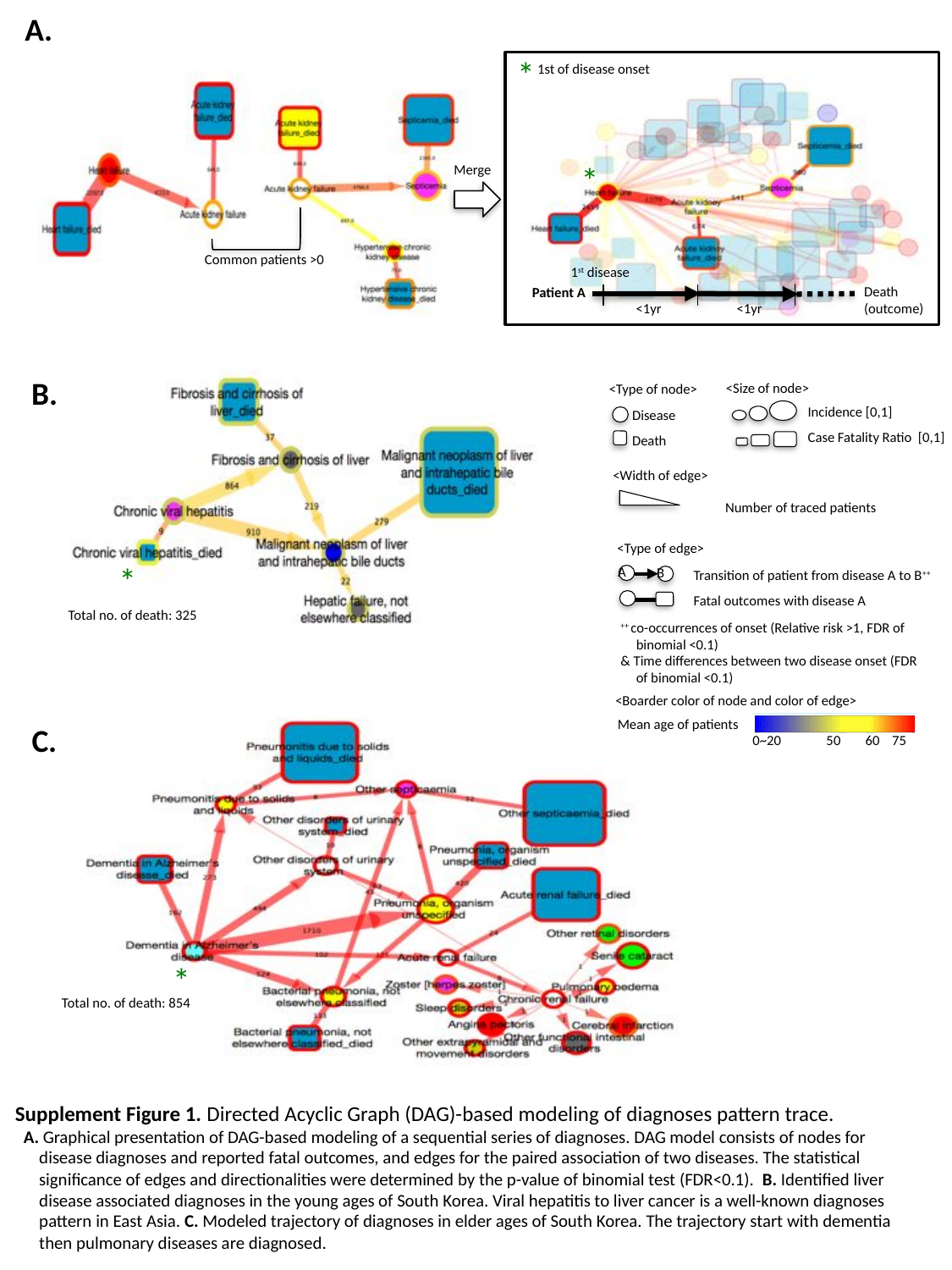

A.
*
1st of disease onset
*
Merge
Common patients >0
1st disease
Death
(outcome)
Patient A
<1yr
<1yr
B.
<Size of node>
<Type of node>
Incidence [0,1]
Case Fatality Ratio [0,1]
Disease
Death
<Width of edge>
Number of traced patients
<Type of edge>
Transition of patient from disease A to B++
Fatal outcomes with disease A
A
B
*
Total no. of death: 325
++ co-occurrences of onset (Relative risk >1, FDR of binomial <0.1)
& Time differences between two disease onset (FDR of binomial <0.1)
<Boarder color of node and color of edge>
Mean age of patients
0~20
60
75
50
C.
*
Total no. of death: 854
Deadliest trajectory for elders (>75 years)
Supplement Figure 1. Directed Acyclic Graph (DAG)-based modeling of diagnoses pattern trace.
A. Graphical presentation of DAG-based modeling of a sequential series of diagnoses. DAG model consists of nodes for disease diagnoses and reported fatal outcomes, and edges for the paired association of two diseases. The statistical significance of edges and directionalities were determined by the p-value of binomial test (FDR<0.1). B. Identified liver disease associated diagnoses in the young ages of South Korea. Viral hepatitis to liver cancer is a well-known diagnoses pattern in East Asia. C. Modeled trajectory of diagnoses in elder ages of South Korea. The trajectory start with dementia then pulmonary diseases are diagnosed.
