# Supplementary material for: Identification of a pleiotropic effect of ADIPOQ on cardiac dysfunction and Alzheimer’s disease based on genetic evidence and health care records
Source: Transl Psychiatry. 2022 Sep 16;12:389. doi: 10.1038/s41398-022-02144-0 (PMC9481623; doi:10.1038/s41398-022-02144-0)
Supplement: Supplementary file 5 — Supplementary Figure 2 [file 41398_2022_2144_MOESM5_ESM.pptx]

## Slide 1
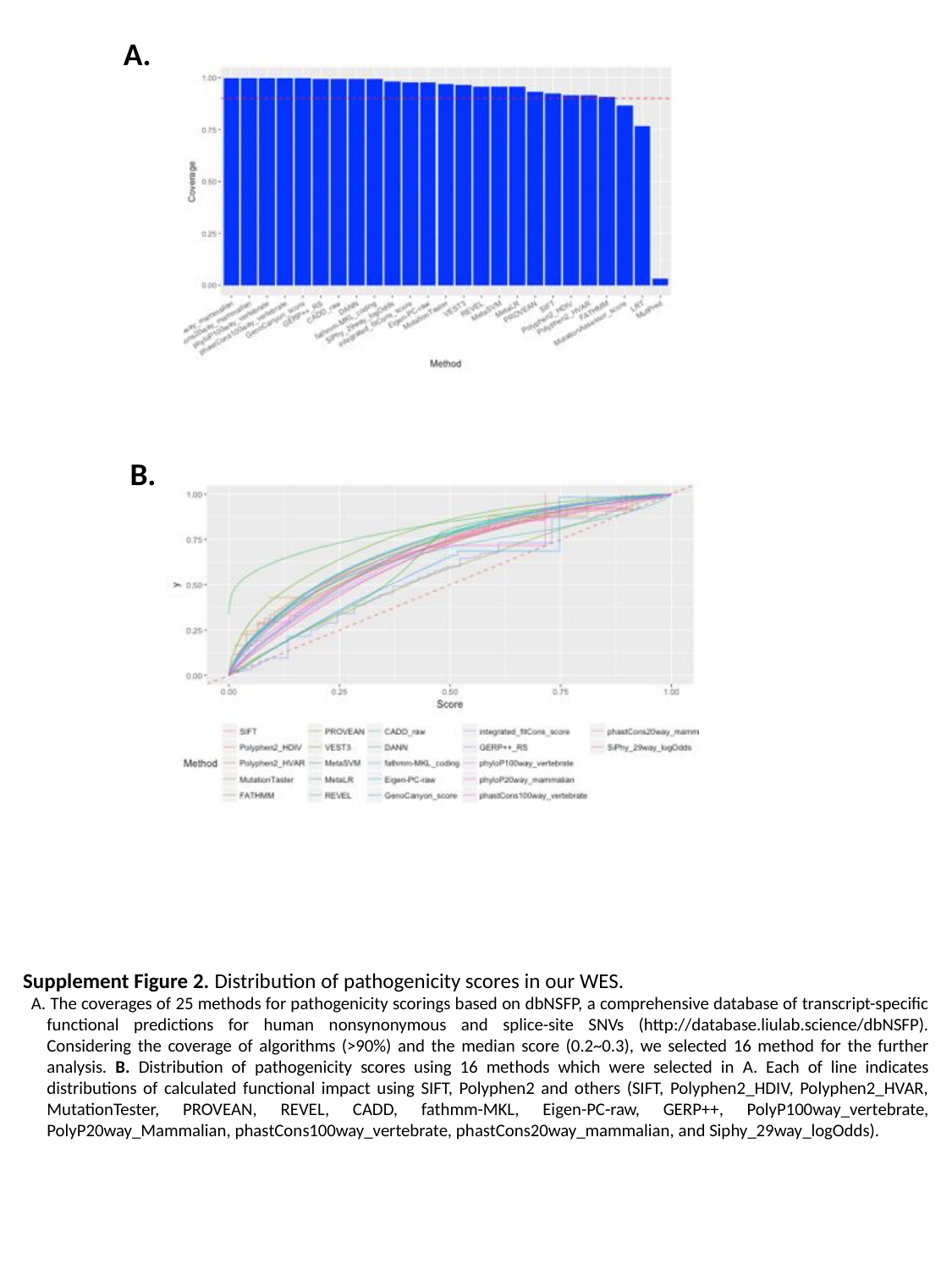

A.
B.
Supplement Figure 2. Distribution of pathogenicity scores in our WES.
A. The coverages of 25 methods for pathogenicity scorings based on dbNSFP, a comprehensive database of transcript-specific functional predictions for human nonsynonymous and splice-site SNVs (http://database.liulab.science/dbNSFP). Considering the coverage of algorithms (>90%) and the median score (0.2~0.3), we selected 16 method for the further analysis. B. Distribution of pathogenicity scores using 16 methods which were selected in A. Each of line indicates distributions of calculated functional impact using SIFT, Polyphen2 and others (SIFT, Polyphen2_HDIV, Polyphen2_HVAR, MutationTester, PROVEAN, REVEL, CADD, fathmm-MKL, Eigen-PC-raw, GERP++, PolyP100way_vertebrate, PolyP20way_Mammalian, phastCons100way_vertebrate, phastCons20way_mammalian, and Siphy_29way_logOdds).
