# Supplementary material for: Identification of a pleiotropic effect of ADIPOQ on cardiac dysfunction and Alzheimer’s disease based on genetic evidence and health care records
Source: Transl Psychiatry. 2022 Sep 16;12:389. doi: 10.1038/s41398-022-02144-0 (PMC9481623; doi:10.1038/s41398-022-02144-0)
Supplement: Supplementary file 6 — Supplementary Figure 3 [file 41398_2022_2144_MOESM6_ESM.pptx]

## Slide 1
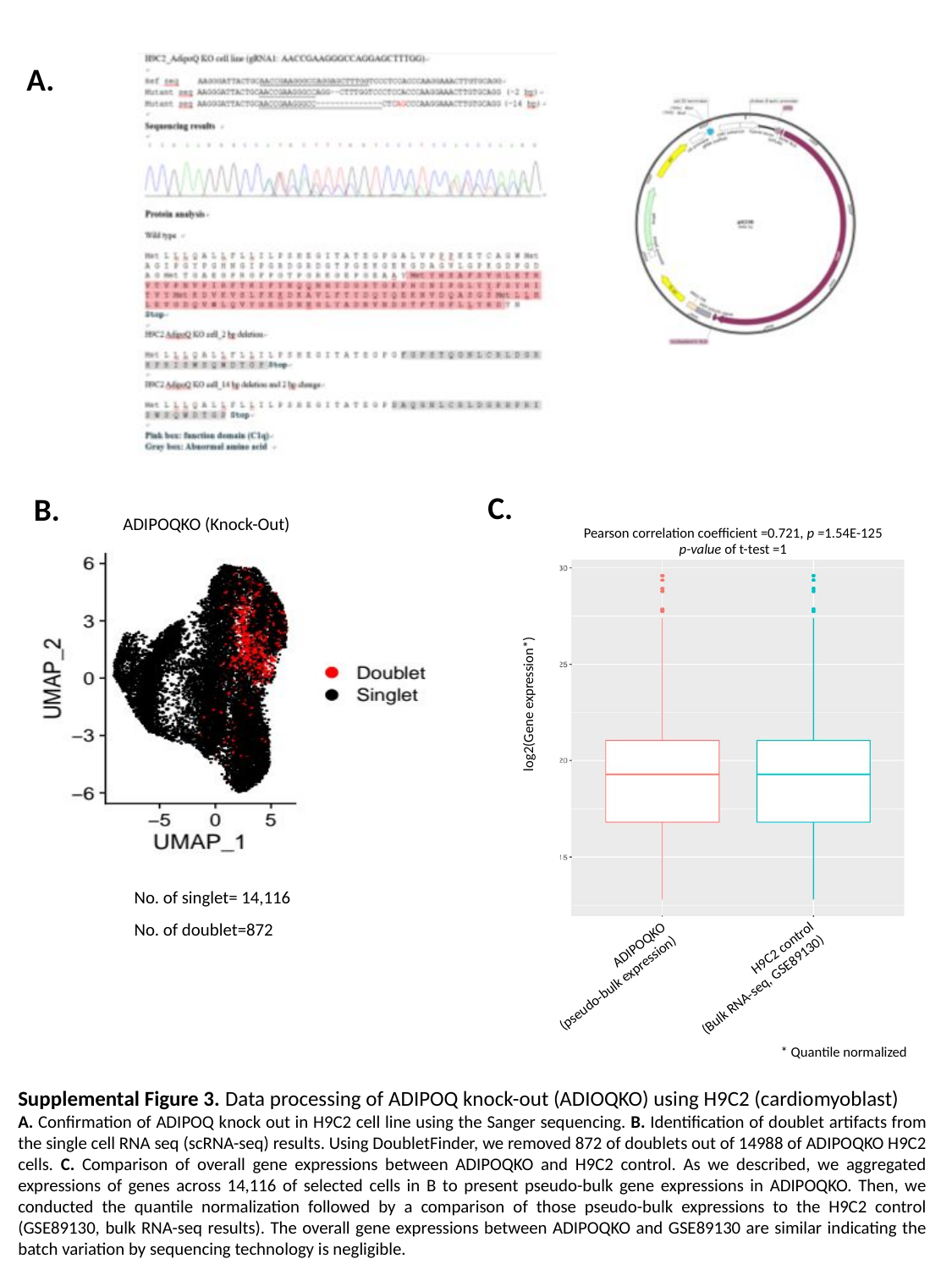

A.
C.
B.
ADIPOQKO (Knock-Out)
Pearson correlation coefficient =0.721, p =1.54E-125
p-value of t-test =1
log2(Gene expression*)
No. of singlet= 14,116
No. of doublet=872
ADIPOQKO
(pseudo-bulk expression)
H9C2 control
(Bulk RNA-seq, GSE89130)
* Quantile normalized
Supplemental Figure 3. Data processing of ADIPOQ knock-out (ADIOQKO) using H9C2 (cardiomyoblast)
A. Confirmation of ADIPOQ knock out in H9C2 cell line using the Sanger sequencing. B. Identification of doublet artifacts from the single cell RNA seq (scRNA-seq) results. Using DoubletFinder, we removed 872 of doublets out of 14988 of ADIPOQKO H9C2 cells. C. Comparison of overall gene expressions between ADIPOQKO and H9C2 control. As we described, we aggregated expressions of genes across 14,116 of selected cells in B to present pseudo-bulk gene expressions in ADIPOQKO. Then, we conducted the quantile normalization followed by a comparison of those pseudo-bulk expressions to the H9C2 control (GSE89130, bulk RNA-seq results). The overall gene expressions between ADIPOQKO and GSE89130 are similar indicating the batch variation by sequencing technology is negligible.
