# Supplementary material for: Identification of a pleiotropic effect of ADIPOQ on cardiac dysfunction and Alzheimer’s disease based on genetic evidence and health care records
Source: Transl Psychiatry. 2022 Sep 16;12:389. doi: 10.1038/s41398-022-02144-0 (PMC9481623; doi:10.1038/s41398-022-02144-0)
Supplement: Supplementary file 8 — Supplementary Table 1 [file 41398_2022_2144_MOESM8_ESM.docx]

**Additional file 8.** Statistics of selected individuals from the UK Biobank

| **Features** | **Major allele (G)**  **(ADIPOQ c.268 G)**  **n = 276** | **Mino allele (A)**  **(ADIPOQ c.268 A)**  **n = 69** | ***p*-value (*t* test)** |
| --- | --- | --- | --- |
| Sex  Male  Female | 133  143 | 34  35 | 0.81 |
| Age (y) | 53.95 ± 7.5 | 53.45 ± 7.9 | 0.63 |
| Height (cm) | 171.11 ± 9.3 | 171.26 ± 8.5 | 0.89 |
| Weight (kg) | 74.85 ± 14.17 | 76.21 ± 11.3 | 0.39 |
| Body Mass Index (BMI) | 25.65 ± 3.8 | 26.08 ± 3.3 | 0.34 |
