# Supplementary material for: Identification of a pleiotropic effect of ADIPOQ on cardiac dysfunction and Alzheimer’s disease based on genetic evidence and health care records
Source: Transl Psychiatry. 2022 Sep 16;12:389. doi: 10.1038/s41398-022-02144-0 (PMC9481623; doi:10.1038/s41398-022-02144-0)
Supplement: Supplementary file 9 — Supplementary Figure 4 [file 41398_2022_2144_MOESM9_ESM.pptx]

## Slide 1
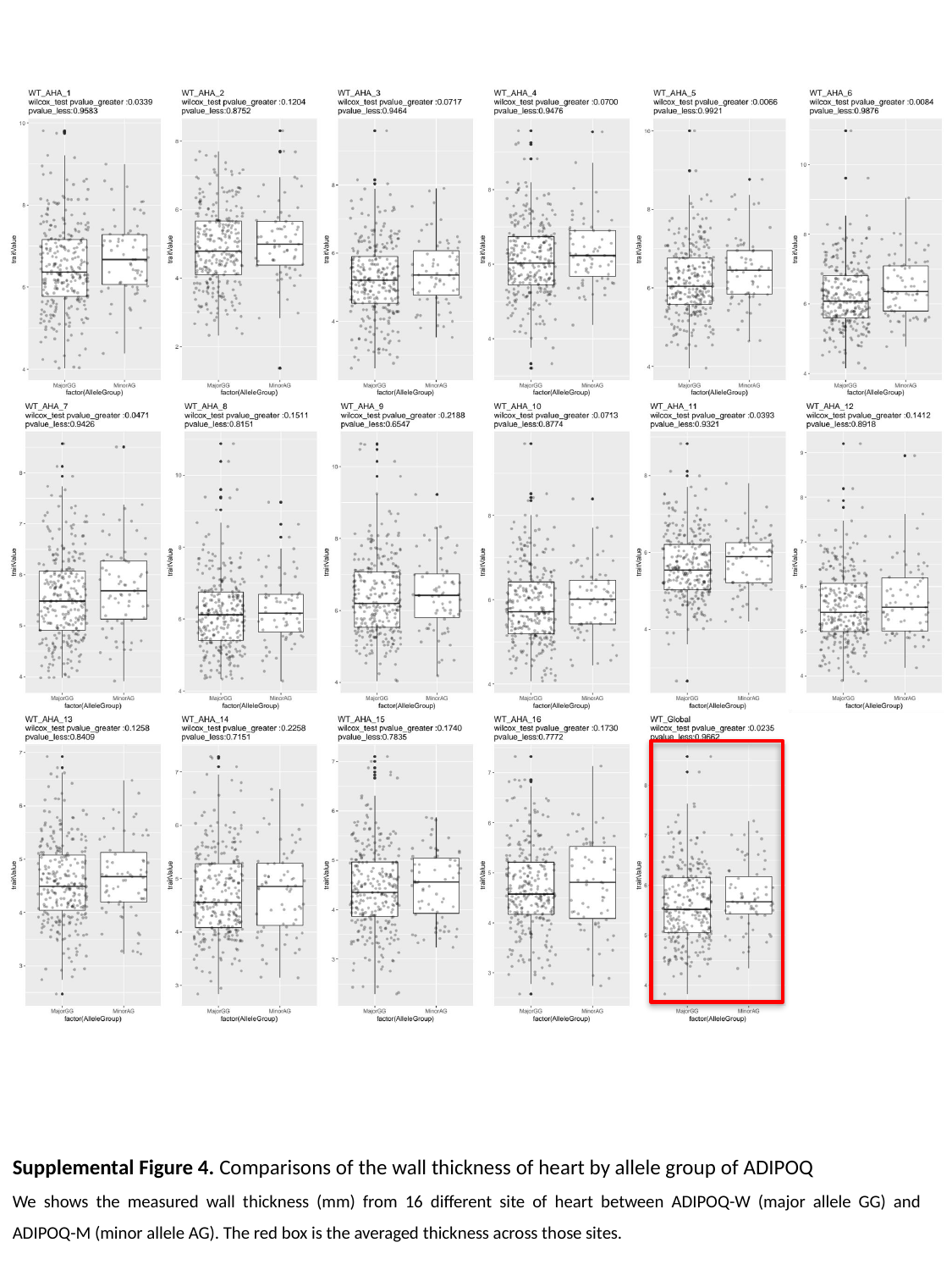

Supplemental Figure 4. Comparisons of the wall thickness of heart by allele group of ADIPOQ
We shows the measured wall thickness (mm) from 16 different site of heart between ADIPOQ-W (major allele GG) and ADIPOQ-M (minor allele AG). The red box is the averaged thickness across those sites.
